# The effects of park-based interventions on health: a systematic review protocol

**DOI:** 10.1186/s13643-020-01396-5

**Published:** 2020-06-08

**Authors:** Deshira D. Wallace, Kathryn P. Derose, Bing Han, Deborah A. Cohen

**Affiliations:** 1 Gillings School of Global Public Health, University of North Carolina at Chapel Hill, 170 Rosenau Hall, CB#7400, 135 Dauer Drive, Chapel Hill, NC 27599 USA; 2 RAND Corporation, 1776 Main Street, Santa Monica, CA 90401 USA

**Keywords:** Parks, Park-based interventions, Physical activity, Physical health, Mental health

## Abstract

**Background:**

Public parks serve as spaces within neighborhoods for encouraging a variety of physical and mental health-related behaviors. Over the past decade, there have been a number of interventions conducted in public parks, often aimed at improving an aspect of mental or physical health. A common type of park-based interventions is aimed at increasing physical activity among adults and children.

**Methods:**

We will conduct a systematic review of peer-reviewed articles on the effects of park-based interventions on physical and mental health outcomes of adults and youth (children and adolescents). An electronic search will be conducted in four electronic databases: Web of Science, PubMed/MEDLINE, Cochrane Library, and Scopus. Manual hand-searching of reference lists from studies identified as relevant by experts and of systematic reviews resulting from the search strategy will be conducted to further identify articles of interest. Inclusion criteria are peer-reviewed, quantitative studies, studies detailing an intervention conducted in a park setting, which was at the person-level or place-level, and studies published in English or Spanish. A three-stage approach will be used to screen title and abstracts and full-text articles against the inclusion and exclusion criteria and, lastly, extract data from eligible studies. Study quality will be assessed by the Cochrane Risk of Bias and the Community Guide’s Guide to Community Preventive Services tools. Extracted data will be summarized narratively and meta-analysis will be conducted, if appropriate.

**Discussion:**

We aim to find relevant studies proving evidence for park-based intervention studies and their effects on health-related outcomes for youth and adults. The evidence obtained from the included studies will help guide future studies on park-based interventions. The study results will be submitted to a peer-reviewed journal for electronic dissemination.

**Systematic review registration:**

Systematic review registration: PROSPERO CRD42018109165.

## Background

The built environment consists of human-made spaces where people “live, work, and recreate on a day-to-day basis” [1]. These environmental spaces have been planned for human utilization; however, specifically how they are best utilized to promote health requires additional investigation [2]. The built environment is defined broadly as green spaces, parks, neighborhood sidewalks, traffic flow, cleanliness, and maintenance of public spaces [2–4]. In general, multi-level studies that have examined the role of the built environment on health have found an association between access to and quality of physical spaces on reduced feelings of distress [5, 6], engagement with physical activity [7], and perceived social connectedness [5, 8].

The built environment, in particular parks, can impact physical activity through walkability and mobility [2]. Physical activity behaviors are associated with health benefits; however, the majority of youths (children and adolescents) (76%) and adults (80%) in the USA do not meet the current physical activity recommendations [9]. Interventions in public parks aimed to improve health is of particular interest as these areas can be modified to reduce barriers for engagement in healthy behaviors at a community level [10]. Public parks are generally accessible to individuals as an estimated 75% of people in the USA live within walking distance of a park; however, accessibility varies significantly by region and demographics [11, 12]. Demographically, a National Recreation and Parks Association survey found that most respondents that lived near a park were of Latino and non-White descent; however, this survey was limited in capturing whether or not these were high-quality parks [13]. Perceptions of parks being of high quality has been shown to be a driver of park use across race and ethnicity [13]. A limited number of studies report park use by race and ethnicity; therefore, the conclusions on park usage are mixed. A systematic review by Joseph and Maddock reported that park-based observational studies typically had a disproportionate share of White park users as compared to non-White park users in the USA [14, 15]. However, other studies have supported that there are few differences in park use among racial and ethnic groups, yet there may be differences in how parks are used for physical activity [11].

In addition, nearly 60% of US adults of all socioeconomic backgrounds use services provided by local recreation departments at least once in their lifetime, with fewer participants (32%) reporting using local recreation and services in the last year [12]. These studies suggest that availability and accessibility to public parks may be relatively similar across the socioeconomic spectrum. Park usage is not equivalent, however, to engagement in health-related activities. Observational studies to date have shown that public parks are often under-utilized, in particular for physical activity [16–20]. Parks have also been linked to mental health-related outcomes. Sturm and Cohen found that mental health, measured using the 5-item Mental Health Inventory, was related to park distance such that residing closer to parks was associated with better mental health scores compared to residing further away from parks [21]. Additionally, Wood et al. found that the presence, accessibility, and size of public spaces were associated with positive mental health-related outcomes [22].

Thus, recent studies have aimed to increase physical activity for park users in multiple ways, by [1] changing the physical structure of the parks (i.e., adding walking trails) to facilitate physical activity (place-level interventions) and/or [2] providing free or low-cost group wellness programs in parks (person-level intervention) [20]. As parks serve as a potential site for increasing physical activity and overall wellness, understanding the changes to park characteristics and/or park programming that improve the amount of park users as well as the wellness for park users is necessary. Previous systematic reviews involving parks have focused on observational studies only (i.e., examining park use but in the absence of an intervention) [14, 15] on the existing physical environment in and near parks (e.g., park facilities, neighborhood walkability) [23] or on park-based interventions with a specific subpopulation—e.g., individuals with disabilities [24]. However, as of this time there are no published systematic reviews that have examined the effects of park-based interventions (both place-level and person-level interventions) on physical and mental health-related outcomes in a general population.

Therefore, the objective of this systematic review is to identify person-level and place-level interventions conducted in parks that targeted health-related outcomes, including physical activity and mental health, using data from published peer-reviewed articles. We will evaluate studies involving adults and youth (children and adolescents). To our knowledge, this is the first systematic review of its kind that broadly summarizes multiple forms of park-based interventions that target health-related outcomes for youth and/or adults.

## Methods

### Design and registration

The design of this research study will be a systematic review. This systematic review will adopt and follow the reporting guidelines and criteria set in the Preferred Reporting Items for Systematic Reviews (PRISMA) statement and standard in the systematic review protocol reporting (PRISMA-P) [25]. The protocol is registered with the International Prospective Register of Systematic Reviews (PROSPERO), CRD42018109165.

### Inclusion and exclusion criteria for considering studies

For this systematic review, we are interested in park-based interventions. Parks are considered as open spaces accessible to the general public, in urban, rural, or suburban areas that are managed by government (i.e., city or state parks and recreation department) or non-governmental entities. The inclusion criteria we will evaluate articles by are [1] peer-reviewed (published only), [2] published in English or Spanish, [3] evaluated physical and mental health-related outcomes, and [4] described an intervention conducted in a park accessible to the larger community (i.e., not a schoolyard restricted to the school’s students). Furthermore, we will focus on empirical research studies using quantitative study designs and research methods. In terms of study designs, intervention types including randomized control trials, cluster-randomized trials, and quasi-experimental designs with or without comparison groups will be included. In addition, intervention types can be at the person-level or at the park- or place-level. As for research methods, we will include studies that present person-level or place-level outcomes.

While qualitative indicators may be present in the article, if the study used a mixed-methods design, we will only extract the quantitative data. We will exclude abstracts, dissertation/theses, blogs, newsletters, organization documents and government reports, book and book chapters, conference proceedings, case reports, and comments. In addition, we will exclude studies evaluating Public Open Space (e.g., plazas, monuments, memorial sites) which are not conducive to physical activity, studies assessing neighborhood-level characteristics (sidewalks), and studies conducted in national parks.

### Populations of interest and exposure measures

This review will include articles that reported person-level interventions that involved the comparison of groups that received a health-related intervention at a park (or prescribed park use) compared to those who did not. Additionally, one-arm studies that evaluated health-related outcomes for the cohort at pretest and post-test will be included. We are also interested in place-level interventions held at parks that compared park use and related health behaviors or health status before and after an environmental change, such as the addition of equipment, shade, or trails in the park. Studies across age groups will be included to characterize interventions in parks for children and adults. There will not be any restrictions for the gender or geographic location of the study participants.

As for exposure measures, the exposures of interest are at two levels according to level of the intervention. For person-level interventions, the exposure will be the assignment to a group (i.e., intervention compared to control) or engagement in a program. For place-level interventions, the exposure if the park or parks that have been manipulated.

### Outcome measures

All health-related outcomes are eligible for this review. We plan to extract two levels of outcomes, i.e., person-level and place-level. Person-level health-related outcomes are those that the studies authors measured from individuals and can include health behaviors, such as physical activity (e.g., moderate-to-vigorous physical activity, sedentary behavior), strength, balance, stress, fatigue, mental well-being, and body mass index (BMI). Place-level health-related outcomes are those calculated at the aggregate level (i.e., park level) from an observer. Examples of park-level outcomes are park utilization and aggregate physical activity intensity. We are keeping the outcomes broad to capture the interventions conducted in parks.

### Search strategy

The following databases will be used to search for relevant peer-reviewed publications, specifically interventions or empirical studies found in Web of Science, PubMed/MEDLINE, Cochrane Library, and Scopus. Manual hand-searching of reference lists from studies identified as relevant by experts and of systematic reviews resulting from the search strategy will be conducted to further identify articles of interest. The lead author will consult with experts in the field to identify any other relevant articles to further fine-tune the search strategy. The search strategy will be limited to studies published in English or Spanish, as that is the capacity of the authors and covers the majority of published articles. The search strategy will be applied through September 2019; however, we plan to re-apply the search strategy prior to publication to ensure the results are not outdated. The search terms for the review focus on three domains, specifically location (e.g., parks, built environment), health-related outcomes (e.g., physical activity, sedentary), and study design (e.g., RCT, observation, SOPARC).

The proposed PubMed/MEDLINE search strategy is described in the Appendix.

The PubMed/MEDLINE search strategy will be adapted to the syntax of the Cochrane Library, Web of Science, and Scope.

The search strategy will be conducted in conjunction with a research librarian who has an expertise in systematic reviews. We will work with the research librarian to fine-tune the search strategy. In addition, we will review the results of the literature search to against known publications that fit our criteria to check for the search strategy’s sensitivity and make any adjustments, if necessary.

All records will be downloaded and deduplicated in EndNote (V8). The deduplicated list of records will be imported into Covidence, an online, systematic review platform that allows for screening of records by multiple users.

### Identification and selection of studies

**Fig. 1:**
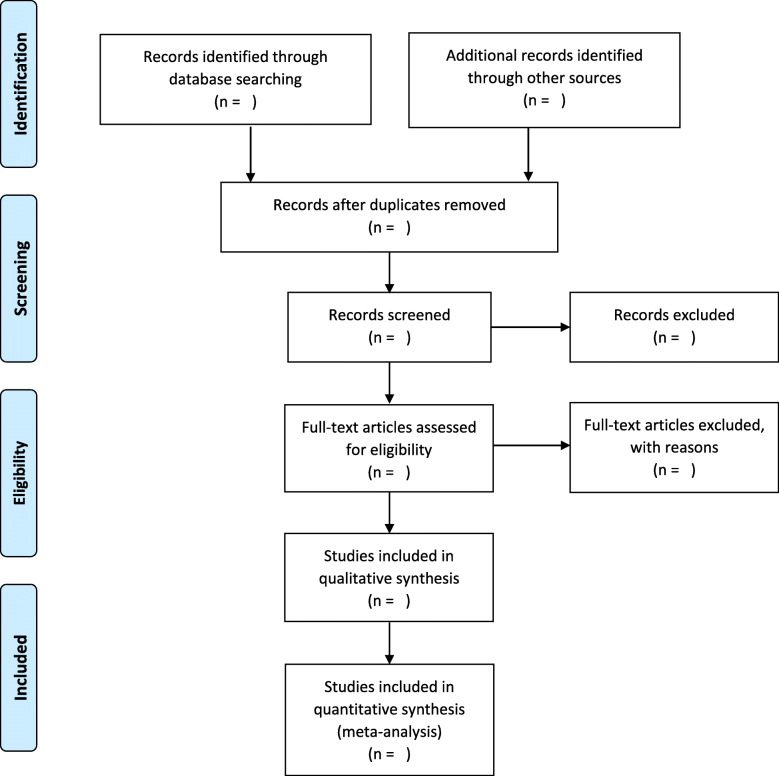
Summary of studies selection procedure using PRISMA

Firstly, two screeners trained on the inclusion criteria and experienced in systematic reviews will independently screen relevant studies (title, abstract, keywords) in the Covidence system. Covidence keeps track of excluded and included studies as well as any discrepancies that require additional review by the senior authors. Secondly, two screeners will independently screen the full text of articles in the Covidence system. During the full-text screening, rationale for excluding studies will be recorded in the Covidence system. Any discrepancies during the full-text screening will be reviewed by the senior author for reconciliation. The final list of studies will be reviewed by the lead author to ensure quality assurance. Per PRISMA guidelines, a flow diagram will be developed to show the process of study selection at different phases (Fig. 1).

### Data extraction and management

The final list of studies will undergo data extraction using the Community Guide’s *Guide to Community Preventive Services* tool [26]. This tool contains 55 questions; however, we will adapt and add questions to account for the needs of this extraction for a total of 62 questions. The types of information extracted will include [1] descriptive information (e.g., study reference, the purpose of the study, how the intervention was being delivered, study design, geographic location, and study site) [2]; the study population (e.g., eligibility criteria, demographic characteristics, sampling method, sample size, attrition details) [3]; results (estimates, significance, interpretation); and [4] study quality. These questions will be transcribed to an online survey platform, Qualtrics, and will include both structured interview and open-response options. Once trained on the tool, two co-authors will independently extract study information. After data extraction is complete, two co-authors will conduct a quality assurance check to ensure all data were accurately extracted.

### Risk of bias

Two reviewers will independently assess the included studies for bias. For randomized controlled trials (RCTs) we will follow the Cochrane Handbook for Systematic Reviews of Intervention to assess the risk of bias [27]. We will assess the risk of bias for RCTs for the following domains: attrition (incomplete outcome data), detection (masking of outcome), performance (masking of participants and personnel), selection (allocation, concealment), and other unspecified bias.

The quality of each eligible non-randomized controlled trials will be assessed using the validated Guide to Community Preventive Services [26]. The key domains of the Guide to Community Preventive Services tool used to determine the quality of the studies are description of the study, sampling type, measurement, analysis, interpretation of results, and other details. Quality for all included studies will be assessed by the first author by reviewing extracted data for completeness and accuracy. Differences in quality assessment will be resolved by all authors through discussion.

### Data analysis and reporting the findings

The data synthesis will include a descriptive summary of the study characteristics. We will evaluate and summarize the methodological quality of the included studies. A table on empirical outcomes found by intervention type and primary outcome will be presented as relative risks, odds ratios, or risk difference for dichotomous outcomes or mean or mean differences for continuous outcomes. Longitudinal studies will be presented with effect sizes of the change in the health-related outcomes over time. Significance values in either *p* values or confidence intervals will be presented if available.

Subgroup analyses that are relevant for this review include comparing adults and youth studies, studies comprised of majority White compared to non-White participants, comparing study outcomes in low-income areas compared to study outcomes in middle- and high-income areas, and comparing person-level and place-level study designs.

Meta-analysis will only be considered when the included studies are sufficiently homogenous in terms of study design, participants, interventions, and outcomes to provide meaningful summary measures. In addition, meta-analysis will also be considered if there are at least two studies available for comparison. If a meta-analysis is appropriate, we will perform a meta-analysis using a random-effects model and calculate pooled effect sizes for each outcome.

For all other health-related outcomes, no quantitative synthesis will be performed and the coefficients of each reported health-related outcome will be described at the study level.

## Discussion

The review is aimed at identifying the available evidence-based park-based interventions that improve health-related outcomes for youth and adults. For place-level studies, we are interested in interventions that physically changed components at the park to encourage or discourage behaviors as a means of improving health. We are also interested in person-level studies, which are cohort interventions that promoted engagement at parks (e.g., exercise groups). Despite the recognition of place-level, structural interventions facilitating health-promoting behaviors and outcomes, there has not been a formal review of what these interventions entail, whom they target, and what health-related outcomes they targeted. This review will identify common measures for assessing engagement in parks or with park facilities by participants in these studies. These results will also elucidate the associations observed between park engagement and various health-related outcomes, or for longitudinal, randomized-control trials, identify the causality between engagement and health-related outcomes. The implications of reviewing both place-level and person-level studies are that evaluating which health behaviors and outcomes change and the extent of those changes occur can inform public health intervention components to target key health behaviors and health-related outcomes. The findings of these studies will be of interest to researchers who implement or evaluate environmental interventions, as it will highlight gaps in evidence that may require further investigation.

### Limitations

We recognize that there are studies for youth that are conducted in school-parks [28–30]; however, these studies will be excluded because the access to these school parks are often restricted to the schools’ students and only operated during school hours. Additional limitations of this systematic review are that we will restrict the search strategy to English and Spanish language, peer-reviewed publications only; therefore, grey literature will be excluded. We are also limiting the search strategy to four databases.

### Plans to dissemination of study results

The study results will be submitted for publication for electronic dissemination. In the final review, any discrepancies between the review and the protocol will be explained. We will ensure that the final manuscript is an accurate and transparent account of the review and that no important aspects of the review will be omitted.

Key stakeholders, including public and private partners intending to develop park-based interventions, were involved in setting the review question and developing the protocol and will assist in interpreting the results. The results of this study will also be presented to stakeholders (practitioners, community representatives) interested in leveraging local parks as sites for health promotion.

## Data Availability

All data generated or analyzed during this study will be included in the published systematic review article.

## Appendix

Appendix 1 Proposed PubMed/MEDLINE search strategy

1. **Domain 1:** ((Park[Title/Abstract] OR parklet*[Title/Abstract] OR built environment[Title/Abstract] OR playfield[Title/Abstract] OR recreation center*[Title/Abstract] OR green space[Title/Abstract] OR fitness zone*[Title/Abstract]))
2. **Domain 2:** AND ((Physical activity[Title/Abstract] OR exercise[Title/Abstract] OR moderate-to-vigorous[Title/Abstract] OR MVPA[Title/Abstract] OR physical health[Title/Abstract] OR mental health[Title/Abstract] OR sedentary[Title/Abstract] OR METs[Title/Abstract] OR metabolic equivalent task[Title/Abstract]))
3. **Domain 3:** AND (intervention*[Title/Abstract] OR RCT[Title/Abstract] OR randomized controlled trial[Title/Abstract] OR SOPARC[Title/Abstract] OR SOPLAY[Title/Abstract] OR system for observing play[Title/Abstract])) OR recreation in communities[Title/Abstract] OR experiment[Title/Abstract] OR program*[Title/Abstract] OR evaluat*[Title/Abstract] OR direct observation[Title/Abstract]))
4. AND (English[Language] OR Spanish[Language])
5. NOT (cattle OR cows OR elephant* OR deer OR boar OR predator* OR leopard* OR national park)
6. NOT (Case reports[Publication Type] OR comment[Publication Type] OR editorial[Publication Type] OR dissertation[Publication Type] OR thesis[Publication Type] OR blog[Publication Type] OR newsletter[Publication Type])

## Abbreviations

PRISMA-P: Preferred Reporting Items for Systematic Review and Meta-Analysis Protocols
PROSPERO: Prospective Register of Systematic Reviews
BMI: Body mass index
MVPA: Moderate to vigorous physical activity
METs: Metabolic equivalent tasks
RCT: Randomized controlled trial
SOPARC: System for Observing Play and Recreation in Communities
SOPLAY: System for Observing Play and Leisure Activity in Youth
